# From Structure to Function and Back Again: A GAN-Guided Diffusion Framework for Generating Clinically Meaningful Multimodal Neuroimaging Data

**DOI:** 10.21203/rs.3.rs-9684909/v1

**Published:** 2026-06-18

**Authors:** Reihaneh Hassanzadeh, Anees Abrol, Hamid Reza Hassanzadeh, Vince D. Calhoun

**Affiliations:** 1 Tri-institutional Center for Translational Research in Neuroimaging and Data Science (TReNDS): Georgia State University, Georgia Institute of Technology, and Emory University, Atlanta, GA, USA; 2 Electrical and Computer Engineering, Georgia Institute of Technology, Atlanta, GA, USA; 3 Courtesy Faculty Appointment, College of Pharmacy, University of Florida, Gainesville, FL, USA

## Abstract

Multimodal brain imaging provides complementary insights into brain structure and function, but its capability is often limited by missing modalities. Traditional imputation and subsampling strategies are computationally simple, but have the risk of introducing bias or discarding valuable samples. Recently, generative models have emerged as powerful alternatives for synthesizing missing modalities. In this study, we introduce a GAN-guided diffusion framework for cross-modality translation, designed to generate both T1-weighted magnetic resonance imaging (MRI) and functional network connectivity (FNC) data. The framework integrates conditional diffusion modeling, adversarial learning, and cycle-consistency, enabling training with both paired and unpaired samples. On Alzheimer’s disease data, our approach outperformed baseline methods, achieving higher peak signal-to-noise ratio (PSNR) (24.95) and structural similarity index measure (SSIM) (0.86) for T1 synthesis, as well as improved correlation with real FNCs (0.65). Furthermore, our results demonstrate that the model captures variability across clinical groups without supervision from diagnostic labels, producing realistic and clinically meaningful synthetic modalities for downstream analysis and biomarker discovery.

## Introduction

Multimodal brain imaging offers a comprehensive view of brain organization by integrating complementary information from different modalities, enabling more accurate analysis of brain structure and function^1^. For example, studies have shown that structural and functional magnetic resonance imaging, while each captures different aspects of brain anatomy and activity, together provide stronger performance in disease classification^2, 3^, biomarker discovery^4–6^, and understanding underlying pathophysiological mechanisms^7^.

Despite the advantages of multimodal imaging, fully paired multimodal datasets remain uncommon in practice, even in large-scale studies, due to several factors such as differences in acquisition protocols across sites, cost and scan-time constraints, and subject intolerance to lengthy scanning sessions.

As a result, multimodal studies often handle missing modalities by discarding incomplete subjects (subsampling) or relying on simple imputation strategies such as mean or zero imputation. However, these approaches can substantially reduce sample sizes and statistical power or introduce bias into the data distribution. To overcome these limitations, generative models offer a promising alternative by synthesizing missing modalities from available ones, enabling the use of both paired and unpaired data while preserving modality-specific variability.

This capability is particularly valuable in neuroimaging studies where some modalities, such as structural MRI, are widely available while others, such as functional MRI, may be missing or inconsistently acquired. A framework capable of learning cross-modal relationships and synthesizing missing data can therefore improve statistical power and broaden the applicability of multimodal analysis methods.

In this study, we employ a GAN-augmented diffusion framework^8^ to generate gray matter T1-weighted MRI and functional network connectivity (FNC) data. The proposed model synthesizes a target modality from a conditioning source modality within a hybrid paired–unpaired cross-modality translation setting. When paired samples are available, the model learns supervised mappings between modalities; when one modality is missing, a synthetic counterpart is generated through a cycle-consistent network, enabling effective training with unpaired data. This design allows the framework to leverage both paired and unpaired datasets, making it well suited for medical imaging scenarios with incomplete multimodal acquisition. We evaluate the framework on Alzheimer’s disease data to demonstrate that the generated samples preserve clinically meaningful variability and disease-related patterns, in contrast to many prior studies that primarily focused on data from healthy individuals.

The main contributions of this work are summarized as follows:

- We implement a GAN-guided diffusion framework capable of learning cross-modality mappings using both paired and unpaired multimodal neuroimaging data.
- We demonstrate effective translation between highly heterogeneous modalities, namely structural MRI and functional connectivity representations.
- We show that the synthesized modalities preserve clinically meaningful variability and diagnostic group differences without requiring diagnostic supervision during training.
- We provide quantitative and qualitative evidence that the proposed framework improves synthesis quality compared with diffusion-only and GAN-based baselines.

## Related Work

Generative models have become a powerful approach for addressing missing modalities in multimodal studies^9,10^. Early work primarily relied on generative adversarial networks (GANs)^11^ for cross-modality synthesis^12–16^, motivated by their ability to learn complex data distributions and generate visually realistic samples. To better control the generation process, studies adopted conditional GANs (cGANs)^15^, where the synthesis is guided by an input modality or auxiliary information. cGAN-based approaches have been widely applied to translation across structural MRI modalities^17,18^, CT-MRI^19^, CT-PET^12^, PET-MRI^20,21^, enabling realistic modality reconstruction and improving downstream tasks including segmentation^22^, registration^23,24^, and medical diagnosis improvement^21^. CycleGAN-based methods^25^ further extended cross-modality synthesis to unpaired training settings, allowing translation between modalities without requiring paired samples^26–28^. Such approaches are particularly attractive for medical imaging scenarios where paired multimodal data are scarce or infeasible to acquire and typically.

Despite their success, GAN-based models are known to suffer from several limitations, including training instability, mode collapse, and limited diversity in generated samples^29^. These issues can be especially problematic in clinical neuroimaging applications, where capturing population-level and subject-specific variability is critical. As a result, recent work has increasingly explored diffusion-based generative models as a more stable alternative for medical image synthesis^30–32^.

Diffusion models have demonstrated improved stability and synthesis fidelity across image generation tasks^33^. In neuroimaging applications, denoising diffusion probabilistic models (DDPMs)^31^ have produced high-quality medical images with strong volumetric consistency, quantitatively outperforming GAN-based approaches^34^. Conditional DDPMs have further demonstrated anatomically consistent brain image synthesis that improves downstream applications such as segmentation and disease classification^35,36^. Compared with GAN-based approaches, diffusion models generally provide more stable training and better coverage of population variability, making them increasingly attractive for medical image synthesis^37^.

Several recent studies have explored hybrid or guided diffusion frameworks to improve cross-modality synthesis. For example, adversarially guided diffusion models incorporate discriminators to encourage realism during the denoising process, bridging the strengths of GANs and diffusion models^8^. Additionally, cycle-guided diffusion models have been introduced to enhance cross-modality MRI synthesis by conditioning paired diffusion processes against each other, leading to improved structural consistency and synthesis quality^38^. While these approaches represent important advances, they typically focus on translating between modalities with similar spatial representations, such as multi-contrast MRI or MRI-to-CT mappings, and often assume the availability of paired training data.

In contrast, translation between structurally heterogeneous modalities, such as structural MRI and functional connectivity representations derived from fMRI, remains relatively underexplored. Moreover, most existing diffusion-based approaches do not explicitly address the realistic scenario in which datasets contain a mixture of paired and unpaired samples across modalities. The proposed framework addresses these limitations by integrating adversarial learning, diffusion modeling, and cycle-consistency within a unified architecture that supports both paired and unpaired training.

## Methods

**Figure 1.**
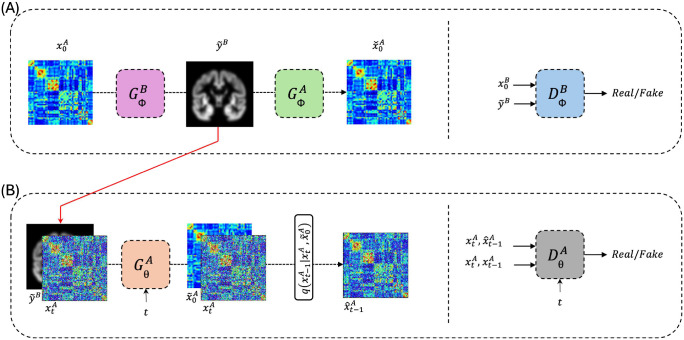
Model overview. (A) **Cycle-consistency path:** $G_{\Phi }^{B}$ translates an input $x_{0}^{A}$ from modality A (e.g., FNC) into modality B (e.g., T1), producing ${\tilde{y}}^{B}$. The second generator $G_{\Phi }^{A}$ maps ${\tilde{y}}^{B}$ back to modality A, yielding a reconstruction ${\overset{ˇ}{x}}_{0}^{A}$. The discriminator $D_{\Phi }^{B}$ evaluates generated ${\tilde{y}}^{B}$ against real samples from modality B. (B) **Conditional diffusion path:** Given a conditioning input (either a real paired sample or a synthetic one from the cycle path), $G_{\theta }^{A}$ predicts denoised samples at intermediate timesteps, progressively refining toward the target modality output. $D_{\theta }^{A}$ enforces adversarial realism in the generated outputs.

An overview of the proposed framework is illustrated in Fig. 1. The model combines a cycle-consistent translation network (Fig. 1(A)) and a conditional diffusion model (Fig. 1(B)) to enable cross-modality synthesis using both paired and unpaired data. When paired samples are available, the diffusion model learns a direct mapping between modalities. When samples are unpaired, a cycle-consistent translation network generates synthetic counterparts that allow the diffusion model to continue training in the absence of paired observations. In this way, the framework jointly leverages paired and unpaired data while maintaining consistency across modalities.

Fig. 1(A) illustrates the cycle-consistency path, which includes two generators, $G_{\phi }^{A}$ and $G_{\phi }^{B}$, and a discriminator $D_{\phi }^{B}$. Generator $G_{\phi }^{B}$ produces a synthetic sample ${\tilde{y}}_{B}$ in domain B from an input $x_{0}^{A}$ in domain A. This generated sample is then translated back into domain A using $G_{\phi }^{A}$, producing a reconstructed sample ${\overset{˘}{x}}_{0}^{A}$. The discriminator $D_{\phi }^{B}$ distinguishes between real samples and generated samples in domain B. This cycle-consistency path is primarily used to generate synthetic modality counterparts when paired samples are unavailable, allowing the diffusion model to be trained on both real paired data and synthetic pairs derived from unpaired samples.

The adversarial objectives used to train the generator and discriminator follow the non-saturating formulation^8^:

(1) $${ℒ}_{G_{\Phi }}=E_{p_{\Phi }\left(y|x_{0}\right)}\left[-\text{log}\left(D_{\Phi }(\tilde{y})\right)\right]$$

(2) $${ℒ}_{D_{\Phi }}=E_{q\left(y|x_{0}\right)}\left[-\text{log}\left(D_{\Phi }(y)\right)\right]+E_{p_{\Phi }\left(y|x_{0}\right)}\left[-\text{log}\left(1-D_{\Phi }(\tilde{y})\right)\right]$$

where $p_{\Phi }\left(y|x_{0}\right)$ denotes the estimated distribution of a target sample conditioned on the source, while $q\left(y|x_{0}\right)$ denotes the true conditional distribution. Table 1 summarizes the main notations used throughout this section for reference.

Fig. 1(B) illustrates the conditional diffusion path used for modality synthesis. Given a conditioning input (either a real paired sample or a synthetic one produced by the cycle path), the generator $G_{\theta }^{A}$ progressively denoises intermediate samples to reconstruct the target modality. Starting from noisy representations, the model iteratively predicts cleaner samples until the final modality estimate is obtained.

The conditional diffusion process incorporates adversarial learning to approximate the reverse diffusion transition $q\left(x_{t-1}|x_{t},y\right)$^39^, where y denotes the conditioning modality. By combining diffusion modeling with adversarial training, the framework encourages realistic intermediate reconstructions while requiring only a small number of diffusion steps (T≈4-10), enabling faster reverse diffusion and efficient sampling compared with standard diffusion models. Discriminator $D_{\theta }^{A}$ enforces adversarial realism by distinguishing between real samples $x_{t-1}\sim q\left(x_{t-1}|x_{t},y\right)$ and generated outputs ${\overset{ˆ}{x}}_{t-1}\sim p_{\theta }\left(x_{t-1}|x_{t},y\right)$ at intermediate diffusion steps.

The corresponding non-saturating loss terms can be expressed as follows:

(3) $${ℒ}_{G_{\theta }}=E_{t,q\left(x_{t}|x_{0},y\right),p_{\theta }\left(x_{t-1}|x_{t},y\right)}\left[-\text{log}\left(D_{\theta }\left({\overset{ˆ}{x}}_{t-1}\right)\right)\right]$$

(4) $${ℒ}_{D_{\theta }}=E_{t,q\left(x_{t}|x_{0},y\right)}\left[E_{q\left(x_{t-1}|x_{t},y\right)}\left[-\text{log}\left(D_{\theta }\left(x_{t-1}\right)\right)\right]+E_{p_{\theta }\left(x_{t-1}|x_{t},y\right)}\left[-\text{log}\left(1-D_{\theta }\left({\overset{ˆ}{x}}_{t-1}\right)\right)\right]+\eta E_{q\left(x_{t-1}|x_{t},y\right)}{\left\|\nabla_{x_{t-1}}D_{\theta }\left(x_{t-1}\right)\right\|}_{2}^{2}\right]$$

where t~U[0,T], and η controls the gradient penalty strength. At each training iteration, a timestep t is sampled uniformly, a noisy sample $x_{t}$ is drawn from the forward diffusion process $q\left(x_{t}|x_{0},y\right)$, and the generator predicts ${\overset{ˆ}{x}}_{t-1}$ according to $p_{\theta }\left(x_{t-1}|x_{t},y\right)$; the expectations in Eq. 3 and 4 therefore average discriminator feedback over these stochastic diffusion transitions encountered during training. Assuming $x_{t}⨿y|x_{0},q\left(x_{t-1}|x_{t},y\right)$ in Eq. 4 can be approximated as $q\left(x_{t-1}|x_{t},x_{0}\right)$. Following^8^, sampling can be performed via:

$$q\left(x_{t-k}|x_{t},x_{0}\right)=𝒩\left(x_{t-1};\overset{‾}{\mu }\left(x_{t},x_{0}\right),\overset{‾}{\gamma }I\right)$$

The estimated distribution $p_{\theta }\left(x_{t-1}|x_{t},y\right)$ is obtained via:

(5) $$p_{\theta }\left(x_{t-1}|x_{t},y\right)≔q\left(x_{t-1}|x_{t},{\overset{ˇ}{x}}_{0}=G_{\theta }\left(x_{t},y,t\right)\right)$$

Furthermore, a cycle consistency loss was applied to the cycle path to ensure that the generated sample ${\tilde{y}}^{A,B}$ could be reconstructed back, imposing a minimal difference between the real sample $x^{A,B}$ and the reconstructed sample ${\overset{ˇ}{x}}_{0}^{A,B}$, and to the diffusion path to enforce a minimal difference between the real sample $x_{0}^{A,B}$ and the synthetic target sample ${\overset{ˆ}{x}}_{0}^{A,B}$:

(6) $${ℒ}_{\text{cyc}}=E_{t,q\left(x_{0}^{A,B}\right),q\left(x_{t}^{A,B}|x_{0}^{A,B}\right)}\sum_{d\in \{A,B\}}\left[\lambda_{\text{cyc}}^{\phi }{\left\|x_{0}^{d}-{\overset{ˇ}{x}}_{0}^{d}\right\|}_{1}+\lambda_{\text{cyc}}^{\theta }{\left\|x_{0}^{d}-{\overset{ˆ}{x}}_{0}^{d}\right\|}_{1}\right]$$

where $\lambda_{\text{cyc}}^{\phi }$ and $\lambda_{\text{cyc}}^{\theta }$ denote the weights of the cycle path and the components of the diffusion path, respectively.

Finally, we introduced a subject-aware loss to learn subject-specific mappings by minimizing the $L_{1}$ distance between real and generated samples by the generators for paired subjects, as computed below:

(7) $${ℒ}_{\text{subj}}=E_{t,q\left(x_{0}^{A,B}\right),q\left(x_{t}^{A,B}|x_{0}^{A,B}\right)}\sum_{d\in \{A,B\}}\left[\lambda_{\text{subj}}^{\phi }{\left\|x_{0}^{d}-{\tilde{x}}^{d}\right\|}_{1}+\lambda_{\text{subj}}^{\theta }{\left\|x_{0}^{d}-{\tilde{x}}_{0}^{d}\right\|}_{1}\right]$$

where $\lambda_{\text{subj}}^{\phi }$ and $\lambda_{\text{subj}}^{\theta }$ control the contributions of the cycle path and diffusion path generators, respectively, to the subject-aware loss.

The total objective combines adversarial, cycle-consistency, and subject-aware losses as:

(8) $${ℒ}_{G}^{\text{total}}=\lambda_{\phi }\sum_{d\in \{A,B\}}{ℒ}_{G_{\phi }^{d}}+\lambda_{\theta }\sum_{d\in \{A,B\}}{ℒ}_{G_{\theta }^{d}}+\lambda_{\text{cyc}}{ℒ}_{\text{cyc}}+\lambda_{\text{subj}}{ℒ}_{\text{subj}}$$

(9) $${ℒ}_{D}^{\text{total}}=\lambda_{\phi }\sum_{d\in \{A,B\}}{ℒ}_{D_{\phi }^{d}}+\lambda_{\theta }\sum_{d\in \{A,B\}}{ℒ}_{D_{\theta }^{d}}$$

where $\lambda_{\phi }$ and $\lambda_{\theta }$ are trade-off weights between the diffusion and cycle-consistency networks, and $\lambda_{\text{cyc}}$ and $\lambda_{\text{subj}}$ control the contributions of the cycle-consistency and subject-aware losses, respectively.

## Experimental Results

### Dataset

In this study, we focus on two modalities: T1-weighted structural MRI and FNC derived from resting-state fMRI. fMRI scans were processed using the NeuroMark independent component analysis (ICA) framework^40^, which extracts 53 reproducible functional brain components. Following ICA and band-pass filtering, the temporal correlations between all pairs of these components were computed to form a 53 × 53 connectivity matrix, referred to as the FNC map. These components are grouped into seven functional network domains: subcortical (SC), auditory (AU), visual (VI), sensorimotor (SM), cognitive control (CC), default mode (DM), and cerebellar (CB)^41^.

We utilized data from the Alzheimer’s Disease Neuroimaging Initiative (ADNI)^42^, which contains 8,372 samples from 1,709 subjects. Table 2 summarizes the distribution of samples and subjects between modalities and diagnostic groups, including control (CN), Alzheimer’s disease (AD), and mild cognitive impairment (MCI). As shown in the last column (FNC and T1), only a small fraction of the dataset contains both T1-weighted MRI and FNC [would update terminology a bit to be more precise: T1-weighted MRI (or structural MRI) and functional MRI/fMRI are modalities, gray matter (GM) and FNC are high dimensional features generated from these modalities] for the same subject, with the FNC modality being the least represented.

### Model Performance

**Figure 2.**
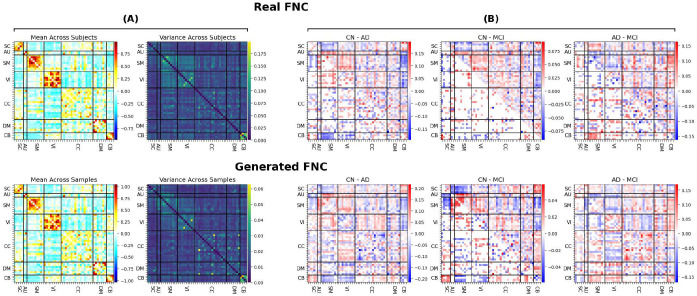
(A) Mean and variance between real and generated FNC maps. (B) Real and generated FNC group differences across diagnostic categories, CN, MCI, and AD. Each matrix shows the difference in mean connectivity values between two groups in the upper triangle, and statistically significant differences (p < 0.05) from two-sample t-tests in the lower triangle.

Fig. 2 demonstrates the ability of the proposed model to generate realistic and diagnostically meaningful FNC maps. Panel (A) shows the mean and variance across subjects, where the generated FNCs closely replicate the functional connectivity structures observed in the real data. The cell-wise variance plots further indicate that variability patterns are largely preserved in the generated FNCs, suggesting that the model does not simply memorize the mean structure but also captures subject-level variability. Panel (B) compares group-level FNC differences across diagnostic categories (CN, MCI, AD) between real and generated samples. The results show the model’s ability to preserve diagnostic contrasts and encode clinically meaningful differences, despite not being guided by diagnostic labels during training.

**Figure 3.**
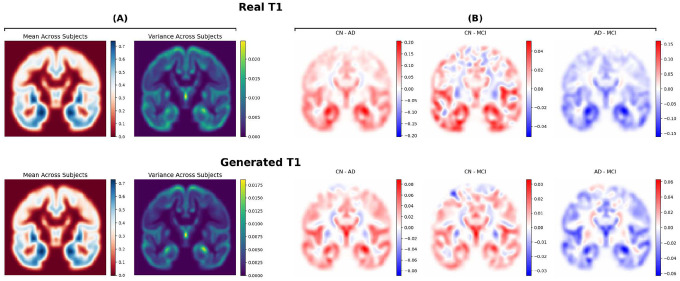
(A) Mean and variance between real and generated T1 images. (B) Real and generated T1 group differences across diagnostic categories, CN, MCI, and AD.

Fig. 3 illustrates the quality and diagnostic relevance of the generated T1-weighted MRI images compared with the real ones. Panel (A) shows the mean and variance across subjects, indicating that the generated images closely match the anatomical structures and variability patterns of the real data. Panel (B) presents group-level differences (CN-AD, CN-MCI, AD-MCI), showing that the generated images not only reproduce the spatial distribution of differences but also capture disease-related changes observed in the real data, such as atrophy in medial temporal and cortical regions.

Table 3 compares the proposed model with several baselines, including a conditional DDPM^31^ and CycleGAN^25^. We evaluated the generated T1 images against their corresponding real ones using the peak signal-to-noise ratio (PSNR) and the structural similarity index (SSIM)^43^, and the generated FNC maps against their corresponding real ones using Pearson correlation (PCorr). The results demonstrate improved performance of our model over the baselines. Specifically, our approach achieved a PSNR of 24.95 and an SSIM of 0.86 in the T1 generation task, as well as a higher correlation of 0.65 in the FNC generation task. These results indicate enhanced image fidelity, structural similarity, and preservation of functional connectivity patterns.

## Discussion

In this work, we presented a GAN-guided diffusion model for cross-modality translation between T1-weighted MRI and functional network connectivity (FNC). Unlike previous studies that focused primarily on translating between structurally similar modalities (e.g., variations of MRI sequences), our model handled two highly heterogeneous representations, structural MRI and connectivity matrices. Through a hybrid training scheme combining adversarial learning and diffusion modeling, the framework reconstructs high-fidelity output while preserving both anatomical detail and functional network structure. The cycle-consistency and subject-aware losses further help capture individual-level variation rather than collapsing toward population averages. As shown by the similarity of mean and variance maps between real and generated data, the synthesized samples preserved both global structure and subject-specific variability. Moreover, the model reproduced clinically relevant group differences between CN, MCI, and AD subjects despite not being trained on diagnostic labels, suggesting that the cross-modal mapping itself embeds disease-related information, including structural atrophy in T1 MRI and patterns of hypo-/hyper-connectivity in FNC.

Quantitative evaluations also highlight the advantages of the proposed framework, with higher PSNR and SSIM for T1 synthesis and substantially stronger correlations for FNC synthesis compared to DDPM and CycleGAN baselines. These improvements suggest greater structural fidelity, more accurate functional organization, and improved robustness to missing modalities, supporting the potential utility of the model for downstream tasks such as data augmentation, classification, and biomarker discovery.

Beyond quantitative performance, the ability of the model to preserve diagnostic contrasts has important scientific implications. Replicated diagnostic differences in both FNC and T1 images indicate that the model learns clinically meaningful patterns that emerge from multimodal relationships rather than explicit supervision. This suggests the potential for analyses where multimodal completeness is limited, such as disease classification, multimodal biomarker identification, or perhaps when acquisition of one modality is not technically feasible. Importantly, synthetic modalities provide an alternative to zero-imputation or subject exclusion policies that often introduce bias or reduce sample sizes in neuroimaging studies.

This study has several limitations. First, the ADNI dataset contains substantial variability due to multi-site and multi-scanner acquisition differences; although the model performed well, generalization to data from unseen sites or different preprocessing pipelines remains to be tested. Second, FNC construction relies on an ICA-based pipeline that imposes its own assumptions on the data. The hybrid GAN–diffusion framework also requires careful tuning of multiple loss terms and hyperparameters. Third, while T1-weighted scans are inherently 3D, we used a representative 2D slice to make training computationally tractable and aligned with the dimensionality of FNC data; this choice may limit the model’s ability to capture certain 3D anatomical relationships.

Future extensions of this work include the incorporation of additional imaging modalities such as PET or EEG to create a unified multimodal diffusion framework capable of synthesizing richer brain representations. Additionally, model interpretability techniques could be applied to understand cross-modal relationships—for example, identifying which structural regions most strongly predict FNC patterns and vice-versa. Another promising direction is to evaluate whether synthesized modalities enhance downstream tasks such as diagnosis, progression prediction, or patient stratification. Finally, scaling the model to full 3D diffusion architectures may enable more detailed anatomical synthesis and better capture disease-specific morphological changes.

## Conclusion

In this study, we introduced a GAN-guided diffusion framework for cross-modality translation in neuroimaging, with a focus on synthesizing T1-weighted MRI and functional network connectivity (FNC) data. By combining conditional diffusion modeling, adversarial training, and cycle consistency, our approach is able to leverage both paired and unpaired data, making it well suited to scenarios with missing modalities. Experiments on Alzheimer’s disease data sets demonstrated that the proposed model outperforms baseline approaches, achieving superior quantitative performance while preserving anatomical detail, functional connectivity structure, and clinically relevant group differences. In general, these results suggest its potential use in downstream tasks such as disease classification and biomarker discovery.

## Supplementary Material

1

## Figures and Tables

**Table 1.** Summary of main notations used in the proposed framework.

Symbol	Description
$x_{0}^{A},x_{0}^{B}$	Clean samples in modalities A and B
$x_{t}$	Noisy sample at diffusion timestep t
${\overset{ˆ}{x}}_{t-1}$	Generated denoised sample at step t-1
${\overset{ˇ}{x}}_{0}$	Reconstructed sample after cycle translation
$\tilde{x}$	Synthetic modality generated by cycle path
$p_{\theta }(\cdot )$	Model-estimated diffusion distribution
q(⋅)	True data diffusion distribution
$G_{\phi }$	Cycle-consistency generators
$G_{\theta }$	Diffusion generators
$D_{\phi }$	Cycle-path discriminator
$D_{\theta }$	Diffusion-path discriminator
y	Conditioning modality input
t	Diffusion timestep index
T	Total diffusion steps
$\lambda_{\text{cyc}}$	Cycle-consistency loss weight
$\lambda_{\text{subj}}$	Subject-aware loss weight

**Table 2.** Data distribution across modalities and diagnostic groups.

AD	# of Samples (# of Subjects)		
	FNC	T1	FNC and T1
CN	411 (214)	2246 (553)	356 (170)
AD	168 (85)	1640 (583)	151 (73)
MCI	465 (203)	3227 (883)	412 (165)

**Table 3.** Comparison of evaluation metric across models.

	FNC − > T1		T1 − > FNC
Model	PSNR	SSIM	PCorr
**Our model**	**24.95**±1.753	**0.86**±0.025	**0.65**±0.011
DDPM	24.48±2.635	0.83±0.097	0.56±0.021
cycle-GAN	23.63±1.426	0.82±0.027	0.49±0.035

## Data Availability

The data used in this study were obtained from the Alzheimer’s Disease Neuroimaging Initiative (ADNI) database (https://adni.loni.usc.edu/). Access to the ADNI dataset is available upon registration and compliance with the ADNI data use agreement. The processed data and code used for this study are available from the corresponding author upon reasonable request.
