# Supplementary material for: From Structure to Function and Back Again: A GAN-Guided Diffusion Framework for Generating Clinically Meaningful Multimodal Neuroimaging Data
Source: Res Sq. 2026 Jun 18:rs.3.rs-9684909. Preprint. [Version 1] doi: 10.21203/rs.3.rs-9684909/v1 (PMC13308360; doi:10.21203/rs.3.rs-9684909/v1)
Supplement: 1 [file NIHPPRS9684909V1-supplement-1.pdf]

Technical Appendices and Supplementary Material

Data Preprocessing

The T1 data were segmented into tissue probability maps for gray matter, white matter, and cerebral spinal fluid using SPM12. The gray matter images were then warped to standard space, modulated and smoothed using a Gaussian kernel with an FWHM = 10mm. The preprocessed gray matter volume images had a dimensionality of 121×145×121 in the voxel space, with a voxel size of 1.5×1.5×1.5 mm^3.

For the resting-state fMRI data sets, we removed the first five time points to ensure signal equilibrium and adaptation of subjects to scanner noise. Then we performed slice timing correction and rigid body motion correction using the SPM toolbox, followed by warping the images into the standard Montreal Neurological Institute (MNI) template using an echo-planar imaging (EPI) template and the old SPM12 normalization module. Lastly, the data were resampled to 3x3x3 mm^3 isotropic voxels, resulting in image dimensionality of 53x63x52 in the voxel space, and smoothed using a Gaussian kernel with a full width at half maximum of 6mm. We also implemented thorough quality control (QC) on the preprocessed fMRI images to discard the images that exhibited (1) poor correlation with individual and group data masks, (2) markedly briefer scan lengths, and (3) high head motion parameters ($>3^\circ$ rotations and $>$3mm translations).

Implementation Details

Network Architectures

The diffusion generators G_θ^A and G_θ^B were implemented using a UNet backbone[44], each consisting of multiple downsampling and upsampling residual blocks with skip connections, group normalization, and Swish activations. Temporal information is injected at each residual block through a learned projection of the sinusoidal timestep embedding, which is added to the feature activations. The input to each generator is formed by concatenating the noisy target modality with the conditioning modality along the channel dimension. The discriminators D_θ^A and D_θ^B are convolutional networks, each consisting of a series of 4×4 convolutional layers with stride 2, doubling the number of feature maps at each stage, followed by LeakyReLU activation. The conditioning modality is concatenated with the generated or real target along the channel dimension.

The cycle-consistency generators G_Φ^A and G_Φ^B were based on ResNet-style architectures[45] with six residual blocks. Each residual block contains two 3×3 convolutional layers, instance normalization, and ReLU activation, with reflection padding to avoid edge artifacts. The discriminators D_Φ and D_θ are PatchGANs[46], consisting of an initial 4×4 convolution with stride 2 and LeakyReLU, followed by a sequence of stride-2 convolutions with doubling feature channels, and ending with a 4×4 convolution that outputs a grid of real/fake scores.

In baselines, in order to have a robust comparison, the DDPM model used similar network architectures as the diffusion path in our network and CycleGAN used similar network architectures for generators and discriminators.

Symmetry Constraint

For experiments involving the synthesis of FNC matrices, we enforced the inherent symmetry property of functional connectivity. After generating a predicted FNC matrix from the network, we retained only the upper triangular portion (excluding the diagonal) and mirrored it to the lower triangular portion. This step enforces the undirected nature of functional brain networks, reduces the number of independent parameters that must be learned, and improves training stability.

Data Splits

We split the dataset into 60% training, 20% validation, and 20% test sets, while ensuring subject-level separation to prevent data leakage across splits.

Hyperparameter Selection

Hyperparameters were tuned using the validation set to optimize Pearson correlation and structural similarity (SSIM) between generated and real samples. We performed a grid search over learning rates $1\mathrm{e}{-4}, 2\mathrm{e}{-4}, 5\mathrm{e}{-5}$, batch sizes $16, 32$, diffusion timesteps $T \in 4$, $\lambda_{\text{cyc}} \in 0.5, 1, 2$, and $\lambda_{\text{subj}} \in 0.5, 1, 2$, while λ_ϕ and λ_θ were fixed at 1. Training was run for 100 epochs, and

the model from the final epoch was selected for evaluation. For the baselines, the DDPM was trained with $T = 1000$ and a cosine scheduler on the paired data, while CycleGAN was trained on all the data. Both baselines were trained for 100 epochs, with a learning rate of $1\text{e}{-4}$ for CycleGAN and $2\text{e}{-4}$ for DDPM.
